# Supplementary material for: Causal Relationship between Aging and Anorexia Nervosa: A White-Matter-Microstructure-Mediated Mendelian Randomization Analysis
Source: Biomedicines. 2024 Aug 16;12(8):1874. doi: 10.3390/biomedicines12081874 (PMC11351342; doi:10.3390/biomedicines12081874)
Supplement: Supplementary file 1 [file biomedicines-12-01874-s001.zip › Supplementary Figure/Figure S4/Singe SNP plot of MR analysis for TL to ubm-b-1521.pdf]

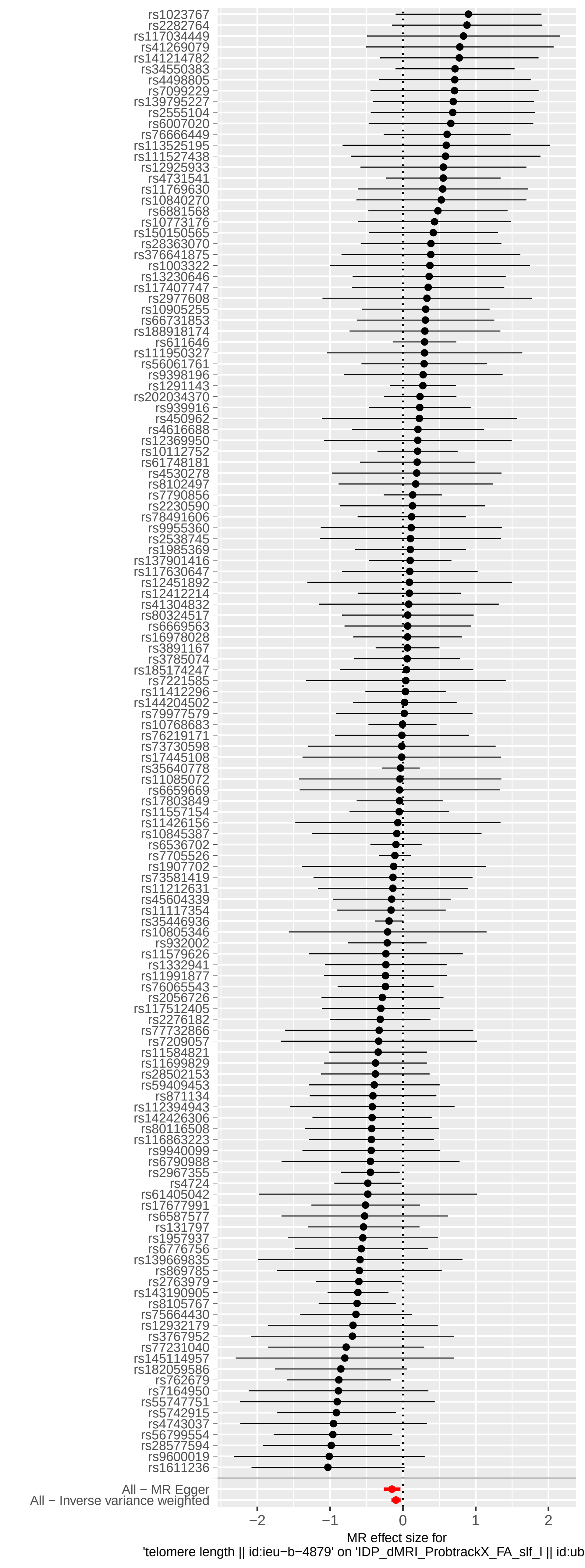

rs1023767
rs2282764
rs117034449
rs41269079
rs141214782
rs34550383
rs4498805
rs7099229
rs139795227
rs2555104
rs6007020
rs76666449
rs113525195
rs111527438
rs12925933
rs4731541
rs11769630
rs10840270
rs6881568
rs10773176
rs150150565
rs28363070
rs376641875
rs1003322
rs13230646
rs117407747
rs2977608
rs10905255
rs66731853
rs188918174
rs611646
rs111950327
rs56061761
rs9398196
rs1291143
rs202034370
rs939916
rs450962
rs4616688
rs12369950
rs10112752
rs61748181
rs4530278
rs8102497
rs7790856
rs2230590
rs78491606
rs9955360
rs2538745
rs1985369
rs137901416
rs117630647
rs12451892
rs12412214
rs41304832
rs80324517
rs6669563
rs16978028
rs3891167
rs3785074
rs185174247
rs7221585
rs11412296
rs144204502
rs79977579
rs10768683
rs76219171
rs73730598
rs17445108
rs35640778
rs11085072
rs6659669
rs17803849
rs11557154
rs11426156
rs10845387
rs6536702
rs7705526
rs1907702
rs73581419
rs11212631
rs45604339
rs11117354
rs35446936
rs10805346
rs932002
rs11579626
rs1332941
rs11991877
rs76065543
rs2056726
rs117512405
rs2276182
rs77732866
rs7209057
rs11584821
rs11699829
rs28502153
rs59409453
rs871134
rs112394943
rs142426306
rs80116508
rs116863223
rs9940099
rs6790988
rs2967355
rs4724
rs61405042
rs17677991
rs6587577
rs131797
rs1957937
rs6776756
rs139669835
rs869785
rs2763979
rs143190905
rs8105767
rs75664430
rs12932179
rs3767952
rs77231040
rs145114957
rs182059586
rs762679
rs7164950
rs55747751
rs5742915
rs4743037
rs56799554
rs28577594
rs9600019
rs1611236
All − MR Egger
All − Inverse variance weighted
−2
−1
0
1
2
MR effect size for
'telomere length || id:ieu−b−4879' on 'IDP_dMRI_ProbtrackX_FA_slf_l || id:ub
